# Neurocognitive features in subgroups of bipolar disorder

**DOI:** 10.1111/bdi.12061

**Published:** 2013-03-25

**Authors:** Sofie Ragnhild Aminoff, Tone Hellvin, Trine Vik Lagerberg, Akiah Ottesen Berg, Ole A Andreassen, Ingrid Melle

**Affiliations:** a KG Jebsen Centre for Psychosis Research, Division of Mental Health and Addiction, Oslo University Hospital and Institute of Clinical Medicine, University of Oslo, Ullevaal HospitalOslo, Norway; b Division of Mental Health Services, Akershus University HospitalLørenskog, Norway

**Keywords:** age at onset, bipolar I disorder, bipolar II disorder, neurocognition, polarity of presenting episode, psychosis

## Abstract

**Objective:**

To examine which subgroups of DSM-IV bipolar disorder (BD) [BD type I (BD-I) or BD type II (BD-II), and subgroups based on history of psychosis, presenting polarity, and age at onset] differentiate best regarding neurocognitive measures.

**Methods:**

A total of 199 patients with BD were characterized by clinical and neurocognitive features. The distribution of subgroups in this sample was: BD-I, 64% and BD-II, 36%; 60% had a history of psychosis; 57% had depression as the presenting polarity; 61% had an early onset of BD, 25% had a mid onset, and 14% had a late onset. We used multivariate regression analyses to assess relationships between neurocognitive variables and clinical subgroups.

**Results:**

Both BD-I diagnosis and elevated presenting polarity were related to impairments in verbal memory, with elevated presenting polarity explaining more of the variance in this cognitive domain (22.5%). History of psychosis and BD-I diagnosis were both related to impairment in semantic fluency, with history of psychosis explaining more of the variance (11.6%).

**Conclusion:**

Poor performance in verbal memory appears to be associated with an elevated presenting polarity, and poor performance in semantic fluency appears to be associated with a lifetime history of psychosis.

Bipolar disorder (BD) is a severe mental disorder with marked heterogeneity in symptomatology, treatment response, clinical course, and outcome 1–4. In addition to the presence of severe mood episodes, the disorder is also associated with varying degrees of psychotic symptoms, neurocognitive impairments, and loss of functioning. This heterogeneity within BD has led to considerable efforts to establish more homogeneous subgroups to use in the search for genetic underpinnings, pathogenic factors, and mechanisms behind treatment response. The DSM-IV comprises two separate subgroups of BD that are exclusively based on differences in their severity of mood elevation; here BD type I (BD-I) is characterized by mania, while BD type II (BD-II) is characterized by hypomania.

Recently, an increased focus on possible BD endophenotypes has emerged from genetic studies. This focus has been emphasized in ongoing work on revisions of the current diagnostic systems (i.e., DSM-5 and ICD-11). In this context, proposals for novel subgroups within the BD spectrum have appeared 3, 5. These include differentiation between psychotic and non-psychotic BD, and between depressive and elevated polarity of the first (or presenting) episode, in addition to groups based on differences in age at onset. However, no studies have so far assessed to what extent these subgroups overlap within one sample, or their ability to discriminate between groups differing in significant characteristics unrelated to those inherent in the grouping procedure, such as cognition.

A recent large international study suggests lifetime prevalence in the general population of 0.6% for BD-I and 0.4% for BD-II 6. Comparative studies indicate that BD-I is associated with psychotic episodes 7 and hospitalizations 8 to a greater extent than BD-II. On the other hand, patients with BD-II are more likely to experience and spend more time in depressive episodes than BD-I patients 2. There are, however, no differences in demographic characteristics, age at onset 9, functional outcome 10, or rate of suicide attempts between BD-I and BD-II 11.

Psychotic symptoms are reported in about 70% of patients with BD-I 12, 13 and 20% of those with BD-II 14, with no gender differences 12, 13. There are indications that psychotic BD constitutes a subgroup with a higher frequency of elevated (manic and hypomanic) mood episodes, more severe mood episodes, more hospitalizations 13, and more cognitive impairments 15, 16 compared to non-psychotic BD. A previous study from our research group also indicated that psychotic BD predicted neurocognitive dysfunction 16 to a larger extent than a BD-I diagnosis, although others do not find this association 17. Patients with non-psychotic BD appear, on the other hand, to have more first-degree relatives with BD 12, 13, more depressive episodes, and a better response to treatment with lithium 13.

For about one-half 18 to two-thirds 19 of cases, the first presenting episode is depression. Studies show either equal sex distribution 19, 20 or a higher frequency of female patients with depressive onset 18. At least in BD-I, the polarity of presenting episode has been shown to be a feature running in families 21, and there are indications that a depressive presenting polarity is associated with earlier age at onset 22, 23, more frequent episodes, and a predominant polarity of depression throughout the course of the disorder 18, 22–24. Others have found similar patterns, but with a later age at onset in depressive presenting polarity 19. Patients with depressive presenting episodes also appear to have longer treatment delays 25, 26 and more suicide attempts 25–27 than patients with elevated or mixed onsets. Polarity of presenting episode could inform treatment, as it may anticipate predominant polarity and thus the most effective medical treatment 28.

The age range for the onset of BD is very wide 29, with no gender differences 30. Previous research has focused on early versus late onset, but recent large multisite studies have identified three potential age at onset-based subgroups with different clinical presentation, across different cultural settings and birth cohorts 31–35: i.e., early onset (mean age at onset ≈ 17 years), intermediate onset (mean age at onset ≈ 26 years), and late onset (mean age at onset ≈ 42 years). The characteristics of the disorder may vary with the age at onset 29, and those with an early onset appear as a separate subgroup with specific clinical manifestations including higher recurrence rates of mood episodes, more elevated episodes at least in BD-I 36, more often depressive onsets 35, more suicide attempts 32, 35, higher risk for comorbid borderline personality disorder 37, higher rates of psychotic symptoms 38, 39, more frequent neurocognitive impairment 40, more BD-I than BD-II 35, and more often a family history of BD 32, 41–44 compared to patients with later onsets.

Thus, there is considerable empirical evidence of subgroups in BD that are associated with differences in clinical course and outcome. There has, however, been limited attention paid to the possibility that the different subgroups describe overlapping phenomena, as indicated by several characteristics common to the suggested groups. Even if we focused here on different aspects or subgroups of a specific disorder, some of the same validating principles should apply here, as for disease entities. Suggested validation criteria for psychiatric illness can be divided into three major categories 45: antecedent validators (family history, demographic, and precipitating factors); concurrent validators (psychological factors derived from, for example, symptom interviews); and predictive validators (relapse, treatment response, and other course descriptions). As the definitions of the proposed subgroups encompass either antecedent and predictive characteristics (age at onset, type, and order of episodes) or antecedent and concurrent clinical syndromes (for BD-I/BD-II and history of psychosis), differentiation based on these characteristics may increase the risk for tautological conclusions. A step forward could be to show subgroup differences in concurrent characteristics that are not directly associated with criteria for subgroup formation. One candidate here is neurocognitive functioning.

Cognitive impairments are present already in the early course of BD 46 and are an important determinant of functional outcome 2, 47. Meta-analyses provide evidence of trait-like neuropsychological deficits in BD involving impairments in attention, processing speed, memory, and executive function 48. Comparative studies suggest that BD-I is characterized by reduced cognitive performance compared to BD-II on executive function 49–51, verbal 16, 50, 51 and working memory 16, and processing speed 50. A recent meta-analytic review also concluded that although BD-II patients are less impaired than BD-I patients on memory and semantic fluency, the overall cognitive impairment in BD-II appears as severe as in BD-I 52. Psychotic BD has been shown to be associated with more impairments than non-psychotic BD, in relation to executive function in general 15, 53–55, and cognitive flexibility in particular 56, 57, as well as on measures of verbal memory 15, 54, 55, working memory 15, 53, 55, 57, and processing speed 15. For other suggested subgroups, the data on cognitive features are rather limited. Early onset may be associated with more severe impairments in verbal memory and processing speed 40. Since neurocognitive impairments are not defining features of clinical subgroups, they can serve as important concurrent validators.

The aim of the current study was to examine to what extent the different suggested ways of subgrouping BD influence cognitive test results in areas of neurocognition previously implicated in BD.

## Materials and methods

### Participants

All participants were consecutively recruited to the ongoing Thematically Organized Psychosis (TOP) Study from outpatient and inpatient units of the three major hospitals in Oslo, Norway, between the years 2003 and 2009. The treating staff asked patients if they were interested in participating in a study of BD, and if so, they were referred to the study. Inclusion criteria for this particular study were age between 17 and 65 years and having a DSM-IV diagnosis of BD-I or BD-II [Total N = 199: BD-I (n = 128), BD-II (n = 71)]. The participants were required to have a Scandinavian language as their first language or have received their compulsory schooling in Scandinavia to assure valid neurocognitive test performance. General exclusion criteria were hospitalization for a head injury, neurological disorder, unstable or uncontrolled medical condition that interferes with brain function, and/or an IQ below 70. The Regional Committee for Medical Research Ethics and the Norwegian Data Inspectorate approved the study, and participants' written, informed consent according to the Declaration of Helsinki was obtained.

The sample consisted of 117 female patients (59%) and 82 male patients (41%); 71 (36%) were employed; 69 (35%) were married or cohabitating. A total of 56 patients (28%) had one or more first-degree relatives with BD (n = 43, 22%), schizophrenia (n = 9, 4%), or both schizophrenia and BD (n = 4, 2%). Lifetime suicide attempt was present in 55 patients (28%). None of these variables differed significantly within any of the four subgroups, except that patients with a depressive presenting polarity were more often single than those with elevated presenting polarity (73% versus 56%, respectively, p *=* 0.017). Mean age for the whole sample was [mean ± standard deviation (SD)] 37 ± 12 years and the median for duration of treatment was one year. There was a significant age difference among the three age at onset groups (mean = 32, 40, and 49 years, respectively, p < 0.001) and between the depressive and elevated presenting polarity groups (mean = 34 versus 39 years, respectively, p = 0.006).

The distribution of the different subgroups was: (i) BD-I (n = 128, 64%) versus BD-II (n = 78, 36%); (ii) psychotic BD (n = 120, 60%) versus non-psychotic BD (n = 78, 40%); (iii) depressive presenting polarity (n = 114, 59%) versus elevated presenting polarity (n = 80, 41%); and (iv) early onset (n = 120, 61%) versus mid onset (n = 49, 25%) and late onset (n = 28, 14%).

### Clinical assessments

Patients were clinically characterized based on a personal interview by trained assessment staff, either medical doctors or clinical psychologists, who had completed the TOP Study's assessment training and reliability program. A good inter-rater reliability for diagnoses was achieved with an overall kappa score of 0.77 (95% confidence interval: 0.60–0.94) 58. Diagnosis was based on the Structured Clinical Interview for DSM-IV Axis I disorders (SCID-I) 59 and information from medical charts. History of psychosis, polarity of presenting episode, and age at onset were determined from the same clinical interview, particularly the SCID information on previous psychotic and mood episodes, and from medical charts. A history of psychosis was defined as having one or more lifetime psychotic episodes. We defined polarity of first presenting episode as the polarity of the first SCID-verified mood episode. Only four patients had a mixed episode as first presenting episode and, due to the low number, they were grouped together with the mania/hypomania as first-episode group. Age at onset was defined as the age of the first SCID-verified mood episode. Age was collapsed into three groups based on results from previous admixture analysis in large samples finding relatively stable age at onset groups in different cultures and birth cohorts: (i) early onset (first episode at <22 years), (ii) mid onset (first episode at between 23 and 34 years), and (iv) late onset (first episode at >35 years) 31–34. Medication status was based on information from interview and medical charts. Current use of mood-stabilizing medication was reported in 119 patients (60%), antidepressants in 80 patients (40%), and antipsychotic medication in 97 (49%). Treatment onset was defined as the first contact with a specialist, regardless of episode polarity. Family history was based on a semi-structured interview asking patients about the presence of BD or schizophrenia in first-degree relatives (parents and siblings). The patient answered whether the diagnosis was probable or sure (as diagnosed by a doctor). We included both. Patients who were adopted or did not know the identity of their father were excluded from this analysis.

The Positive and Negative Symptom Scale (PANSS) was used to measure current psychotic symptoms. The psychosis cut-off was at a level ≥4 on items p1, p3, p5, p6, and/or g9 60. There were no differences in rates of current psychosis between any of the subgroups, apart from the history of psychosis subgroup where 22 patients (19%) had current psychotic symptoms. Current depressive symptoms were measured by the Inventory of Depressive Symptoms-Clinician rated (IDS-C) 61. Here, 91 patients (48%) had no depressive symptoms (IDS ≤ 13), 41 (22%) had possible/mild depression (IDS score 14–21), 32 (17%) had moderate depression (IDS score 22–30), 16 (8%) had severe depression (IDS score 31–38), and 10 (5%) had very severe depression (IDS score ≥39) 62. IDS information was missing for nine patients. Current manic symptoms were rated using the Young Mania Rating Scale (YMRS) 63. Here, 166 patients (84%) had no symptoms of mania (YMRS score ≤7), 29 (15%) had possible/mild mania (YMRS score 8–20), and two (1%) had moderate mania (YMRS score 21–30) 62. YMRS information was missing for two patients. Eighty-six patients (36%) were euthymic (IDS score ≤13 and YMRS score ≤7).

Premorbid adjustment was measured by the Premorbid Adjustment Scale (PAS) 64, and then subdivided into the domains of social and academic adjustment using indices of childhood level and subsequent change, up to the last premorbid period 65. A higher PAS score indicates a lower functioning. Functional and symptomatic levels were assessed with the Global Assessment of Functioning (GAF) Scale, split version 66.

### Neurocognitive assessment

Psychologists trained in standardized neuropsychological testing carried out neurocognitive assessment. A three-hour comprehensive test battery was administered in a fixed order with two breaks with refreshments. Premorbid IQ was assessed with a Norwegian research version of the National Adult Reading Test (NART) 67. There were no differences in premorbid IQ within any of the four subgroups.

Included in this part of the study were neurocognitive tests, which measure cognitive functions sensitive to BD 16. Verbal learning and memory were measured through the Norwegian version of the California Verbal Learning Test (CVLT-II) 68, with repetition errors and intrusions. Working memory was assessed with the Bergen n-back test (2-back) 69, where the number of false positives was subtracted from the number of correct responses. Processing speed was assessed with the Digit Symbol Test [Wechsler Adult Intelligence Scale, Third Revision (WAIS-III) 70]. The executive function domains tested included verbal fluency, verbal interference control, and set shifting. Verbal fluency was measured with the Verbal Fluency Test [Delis–Kaplan Executive Function Scale (D-KEFS)] 71, including both semantic and phonemic fluency, with additional measures of repetition and set loss errors. Verbal interference control was measured through the inhibition trial, and interference set shifting through the inhibition switching trial, of the Color-Word Interference Test (D-KEFS) 71, with additional information about the number of inhibition and inhibition-switching errors. We used raw scores on all tests.

### Statistical analyses

All analyses were performed using The Statistical Package for the Social Sciences (SPSS Inc., Chicago, IL, USA; version 18.0). Bivariate analyses investigating differences between groups (Tables 1–4) used χ² tables for categorical data, Mann–Whitney *U*-tests and Kruskal–Wallis *H-*tests for non-normally distributed continuous data, and *t-*tests and ANOVAs for normally distributed data. The level of statistical significance with Bonferroni correction for multiple testing was set to p ≤ 0.017.

**Table 1:** Clinical and neurocognitive characteristics of patients with bipolar I (BD-I) and bipolar II (BD-II) disorder

	BD-I (n = 128)	BD-II (n = 71)	Test statistics	p-value
Premorbid functioning (PAS)				
Social function childhood, Md [range]	0.5 [0–5]	1 [0–4]	*U* = 5.33	**0.013**^a^
Academic function childhood, Md [range]	1 [0–4.5]	1.5 [0–4.5]	*U* = 5.65	**≤0.001**^a^
Illness course				
Duration of illness, years, mean ± SD	13 ± 11	15 ± 10	*t* = −1.38	0.169
No. of episodes, Md [range]	6 [1–92]	10 [2–252]	*U* = 4.57	**0.001**^a^
No. of depressive episodes, Md [range]	3 [0–47]	4 [1–90]	*U* = 4.75	**0.008**^a^
No. of elevated episodes, Md [range]	2 [1–56]	4 [1–245]	*U* = 4.92	**≤0.001**^a^
CVLT total recall (raw score), mean ± SD	53 ± 11	58 ± 10	*t* = −3.36	**≤0.001**^a^
CVLT long delay, free recall (raw score), Md [range]	13 [4–16]	14 [4–16]	*U* = 5.81	**≤0.001**^a^
CVLT repetitions, Md [range]	4 [0–23]	2.5 [0–15]	*U* = 3.91	0.188
CVLT intrusions, Md [range]	2 [0–54]	2 [0–29]	*U* = 4.38	0.935
Bergen 2-back, Md [range]	14 [−48 to 20]	15 [−10 to 20]	*U* = 4.68	0.560
Digit symbol coding (raw score), mean ± SD	64 ± 17	69 ± 15	*t* = −2.04	**0.043**
D-KEFS: color-word interference				
Inhibition, secs, Md [range]	56 [33–182]	54 [35–187]	*U* = 4.11	0.308
Inhibition/switching, secs, Md [range]	60 [34–186]	59 [37–98]	*U* = 4.10	0.269
D-KEFS: verbal fluency				
Phonetic (raw score), mean ± SD	40 ± 13	42 ± 11	*t* = −0.86	0.389
Semantic (raw score), mean ± SD	41 ± 11	45 ± 10	*t* = −2.65	**0.010**^a^
Repetition errors, Md [range]	2 [0–14]	1 [0–8]	*U* = 3.81	0.077
Set loss errors, Md [range]	1 [0–13]	0 [0–14]	*U* = 3.74	**0.041**
Euthymic, n (%)	57 (44)	14 (21)	χ^2^ = 9.26	**0.002**^a^
Depressive symptoms, IDS, Md [range]	12 [0–51]	19 [2–53]	*U* = 5.47	**≤0.001**^a^
Manic symptoms, YMRS, Md [range]	1.5 [0–24]	2 [0–16]	*U* = 4.83	0.344
GAF (symptom), mean ± SD	57 ± 12	57 ± 8	*t* = 0.22	0.823
GAF (function), mean ± SD	53 ± 12	57 ± 11	*t* = −2.30	**0.017**^a^

Significant results (at p < 0.05) are presented in **bold.**

CVLT = California Verbal Learning Test; D-KEFS = Delis–Kaplan Executive Function System; GAF = Global Assessment of Function; IDS = Inventory of Depressive Symptoms; Md = median; PAS = Premorbid Adjustment Scale; SD = standard deviation; *t* = *t*-test; χ^2^ = chi-square; *U* = Mann–Whitney *U*-test; YMRS = Young Mania Rating Scale.

a Survived Bonferroni correction.

**Table 2:** Clinical and neurocognitive characteristics of patients with bipolar disorder with and without a history of psychosis^a^

	History of psychosis (n = 120)	No history of psychosis (n = 78)	Test statistics	p-value
Premorbid functioning (PAS)				
Social function childhood, Md [range]	1 [0–5]	0.5 [0–4]	*U* = 4.57	0.968
Academic function childhood, Md [range]	1 [0–4.5]	1 [0–4]	*U* = 4.28	0.423
Illness course				
Duration of illness, years, mean ± SD	12 ± 10	16 ± 11	*t* = 2.42	**0.016**^b^
No. of episodes, Md [range]	6 [1–85]	8 [1–252]	*U* = 2.95	**0.041**
No. of depressive episodes, Md [range]	3 [0–42]	4 [0–90]	*U* = 3.56	0.165
No. of elevated episodes, Md [range]	2 [1–60]	4 [1–245]	*U* = 3.01	**0.012**^b^
CVLT total recall (raw score), mean ± SD	53 ± 11	57 ± 11	*t* = 2.07	**0.040**
CVLT long delay, free recall (raw score), Md [range]	13 [4–16]	14 [4–16]	*U* = 3.72	**0.017**^b^
CVLT repetitions, Md [range]	4 [0–23]	3 [0–15]	*U* = 5.33	**0.040**
CVLT intrusions, Md [range]	2 [0–54]	1 [0–29]	*U* = 4.82	0.461
Bergen 2-back, Md [range]	14 [−15 to 20]	15 [−48 to 20]	*U* = 4.45	0.752
Digit symbol coding (raw score), mean ± SD	64 ± 17	68 ± 16	*t* = 1.65	0.101
D-KEFS: color-word interference				
Inhibition, secs, Md [range]	56 [33–182]	52.5 [35–187]	*U* = 5.28	0.103
Inhibition/switching, secs, Md [range]	60 [34–186]	58 [37–98]	*U* = 5.23	0.130
D-KEFS: verbal fluency				
Phonetic (raw score), mean ± SD	41 ± 11	42 ± 12	*t* = 0.74	0.463
Semantic (raw score), mean ± SD	41 ± 11	45 ± 10	*t* = 2.56	**0.011**^b^
Repetition errors, Md [range]	2 [0–14]	1 [0–8]	*U* = 4.92	0.370
Set loss errors, Md [range]	1 [0–13]	0 [0–14]	*U* = 4.99	0.312
Euthymic, n (%)	48 (43)	20 (26)	χ^2^ = 4.67	**0.031**
Depressive symptoms, IDS, Md [range]	12 [0–51]	17 [0–53]	*U* = 5.54	**0.017**^b^
Manic symptoms, YMRS, Md [range]	2 [0–24]	2 [0–15]	*U* = 4.68	0.828
GAF (symptom), mean ± SD	56 ± 13	59 ± 7	*t* = 1.78	0.077
GAF (function), mean ± SD	53 ± 13	58 ± 10	*t* = 2.84	**0.008**^b^

Significant results (at p < 0.05) are presented in **bold.**

CVLT = California Verbal Learning Test; D-KEFS = Delis–Kaplan Executive Function System; GAF = Global Assessment of Function; IDS = Inventory of Depressive Symptoms; Md = median; PAS = Premorbid Adjustment Scale; SD = standard deviation; *t* = *t*-test; χ^2^ = chi-square; *U* = Mann–Whitney *U*-test; YMRS = Young Mania Rating Scale.

a Missing information on one subject.

b Survived Bonferroni correction.

**Table 3:** Clinical and neurocognitive characteristics of patients with bipolar disorder with depressive and elevated polarity of presenting episode^a^

	Elevated (n = 80)	Depressive (n = 114)	Test statistics	p-value
Premorbid functioning (PAS)				
Social function childhood, Md [range]	0.5 [0–5]	1 [0–4]	*U* = 4.25	0.635
Academic function childhood, Md [range]	1 [0–4.5]	1 [0–4.5]	*U* = 4.25	0.642
Illness course				
Duration of illness, mean ± SD	13 ± 11	13 ± 10	*t* = −0.12	0.903
No. of episodes, Md [range]	5.5 [1–252]	8 [2–180]	*U* = 2.87	**0.030**
No. of depressive episodes, Md [range]	2 [0–32]	4 [1–90]	*U* = 2.46	**≤0.001**^b^
No. of elevated episodes, Md [range]	3 [1–245]	3 [1–96]	*U* = 3.92	0.679
CVLT total recall (raw score), mean ± SD	51 ± 12	57 ± 10	*t* = 3.75	**≤0.001**^b^
CVLT long delay, free recall (raw score), Md [range]	12 [4–16]	14 [4–16]	*U* = 3.00	**≤0.001**^b^
CVLT repetitions, Md [range]	4 [0–18]	3 [0–23]	*U* = 4.51	0.772
CVLT intrusions, Md [range]	2 [0–54]	1 [0–17]	*U* = 5.29	**0.017**^b^
Bergen 2-back, Md [range]	14 [−15 to 20]	14 [−48 to 20]	*U* = 4.43	0.929
Digit symbol coding (raw score), mean ± SD	65 ± 18	67 ± 16	*t* = 0.89	0.400
D-KEFS: color-word interference				
Inhibition, secs, Md [range]	57 [39–182]	54 [33–187]	*U* = 5.03	0.180
Inhibition/switching, secs, Md [range]	60 [34–186]	58 [37–112]	*U* = 4.99	0.212
D-KEFS: verbal fluency				
Phonetic (raw score), mean ± SD	40 ± 13	41 ± 12	*t* = 0.64	0.525
Semantic (raw score), mean ± SD	42 ± 11	43 ± 10	*t* = 0.63	0.523
Repetition errors, Md [range]	2 [0–12]	1 [0–14]	*U* = 5.29	**0.028**
Set loss errors, Md [range]	1 [0–13]	0.5 [0–14]	*U* = 4.93	0.202
Euthymic, n (%)	28 (36)	39 (36)	χ^2^ = 0.00	1.000
Depressive symptoms, IDS, Md [range]	14 [0–53]	14 [0–51]	*U* = 4.16	0.811
Manic symptoms, YMRS, Md [range]	2 [0–24]	1 [0–16]	*U* = 4.81	0.366
GAF (symptom), mean ± SD	57 ± 11	58 ± 11	*t* = 0.76	0.450
GAF (function), mean ± SD	53 ± 12	56 ± 12	*t* = 1.90	0.590

Significant results (at p < 0.05) are presented in **bold.**

CVLT = California Verbal Learning Test; D-KEFS = Delis–Kaplan Executive Function System; GAF = Global Assessment of Function; IDS = Inventory of Depressive Symptoms; Md = median; PAS = Premorbid Adjustment Scale; SD = standard deviation; *t* = *t*-test; χ^2^ = chi-square; *U* = Mann–Whitney *U*-test; YMRS = Young Mania Rating Scale.

a Missing information on five subjects.

b Survived Bonferroni correction.

**Table 4:** Clinical and neurocognitive characteristics of patients with bipolar disorder with early, mid, and late onset^a^

	Early onset (n = 120)	Mid onset (n = 49)	Late onset (n = 28)	Test statistics	p-value
Age at onset, years, Md [range]	18 [8–22]	27 [23–34]	41 [35–52]		
Premorbid functioning (PAS)					
Social function childhood, Md [range]	1 [0–4]	0.5 [0–5]	0 [0–2]	*K* = 12.91	**0.002**
Academic function childhood, Md [range]	1 [0–4.5]	1 [0–4.5]	0.5 [0–3.5]	*K* = 16.94	**≤0.001**
Illness course					
Duration of illness, years, mean ± SD	15 ± 10	12 ± 10	7 ± 8	*F* = 8.09	**≤0.001**
No. of episodes, Md [range]	9 [1–252]	5 [1–107]	4 [1–90]	*K* = 12.41	**0.002**^b^
No. of depressive episodes, Md [range]	4 [1–90]	2 [0–40]	2 [0–30]	*K* = 9.38	**0.009**^b^
No. of elevated episodes, Md [range]	3 [1–245]	2 [1–105]	2 [1–60]	*K* = 9.59	**0.008**^b^
CVLT total recall (raw score), mean ± SD	56 ± 10	53 ± 12	51 ± 12	*F* = 2.82	0.062
CVLT long delay, free recall (raw score), Md [range]	14 [4–16]	13 [5–16]	12 [4–16]	*K* = 3.72	0.155
CVLT repetitions, Md [range]	3 [0–23]	4 [0–20]	2 [0–18]	*K* = 0.71	0.700
CVLT intrusions, Md [range]	1 [0–54]	2 [0–10]	2 [0–17]	*K* = 1.37	0.504
Bergen 2-back, Md [range]	15 [−15 to 20]	14 [−48 to 20]	11 [−3 to 20]	*K* = 5.66	0.059
Digit symbol coding (raw score), mean ± SD	67 ± 16	65 ± 19	63 ± 15	*F* = 0.62	0.541
D-KEFS: color-word interference					
Inhibition, secs, Md [range]	53.5 [35–182]	58 [36–89]	56 [33–187]	*K* = 2.35	0.309
Inhibition/switching, secs, Md [range]	58 [37–186]	60 [34–112]	58 [44–90]	*K* = 2.23	0.327
D-KEFS: verbal fluency					
Phonetic (raw score), mean ± SD	41 ± 12	43 ± 13	39 ± 12	*F* = 0.62	0.539
Semantic (raw score), mean ± SD	43 ± 11	43 ± 11	41 ± 8	*F* = 0.75	0.474
Repetition errors, Md [range]	1 [0–14]	2 [0–12]	2 [0–11]	*K* = 7.25	**0.027**
Set loss errors, Md [range]	0 [0–9]	1 [0–13]	1 [0–14]	*K* = 135	0.509
Euthymic, n (%)	35 (31)	17 (36)	15 (56)	χ^2^ = 5.73	0.060
Depressive symptoms, IDS, Md [range]	15 [0–51]	14 [1–53]	8 [0–39]	*K* = 8.76	**0.013**^b^
Manic symptoms, YMRS, Md [range]	2 [0–22]	1 [0–16]	1 [0–24]	*K* = 1.24	0.573
GAF (symptom), mean ± SD	57 ± 11	57 ± 11	59 ± 11	*F* = 0.56	0.574
GAF (function), mean ± SD	55 ± 13	54 ± 12	53 ± 11	*F* = 1.20	0.303

Significant results (at p < 0.05) are presented in **bold.**

CVLT = California Verbal Learning Test; D-KEFS = Delis–Kaplan Executive Function System; *F* = ANOVA; χ^2^ = chi-square; *K* = Kruskal–Wallis test; GAF = Global Assessment of Function; IDS = Inventory of Depressive Symptoms; Md = median; PAS = Premorbid Adjustment Scale; SD = standard deviation; YMRS = Young Mania Rating Scale.

a Missing information on two subjects.

b Survived Bonferroni correction.

To be able to adjust for potential mediators (variables correlated with both subgrouping and outcome variables), we performed bivariate correlation analyses between demographical and clinical variables, measured through Pearson's correlations (*r*). Variables explored were sex, age, education, duration of illness, number of episodes, number of depressive episodes, number of elevated episodes, and level of current symptomatology, such as level of depressive and manic symptoms, and presence of psychotic symptoms.

To explore the effect of potential confounders for the association between group membership and neurocognition, we first performed bivariate correlation analyses between group membership, and neurocognitive, demographic, and clinical variables [Pearson's correlations (*r*)]. Variables explored were sex, age, education, duration of illness, number of episodes, and level of current symptomatology, such as level of depressive and manic symptoms, as well as the presence of psychotic symptoms. We then conducted a series of hierarchical multiple linear regression analyses with neurocognitive variables that showed within-group differences in at least two of the suggested four subgroups as dependents (i.e., verbal memory, verbal learning, and semantic fluency). We used a block-wise forced entry procedure, and in the first block entered variables with significant association with the dependent in bivariate correlations (i.e., age, sex, duration of illness for verbal memory and education, and age for verbal fluency). In the second block we added affective, psychotic symptoms as well as duration of illness and number of episodes, as these theoretically could affect neurocognitive functioning. The third block contained information on subgroup membership for groups that showed neurocognitive differences for the dependents (i.e., BD-I versus BD-II, history of psychosis, presenting episode, and finally age at onset as a continuous variable, respectively).

## Results

### Group differences in patient characteristics

#### Diagnostic subgroup (BD-I versus BD-II)

Patients with a BD-I diagnosis had lower PAS childhood scores and fewer mood episodes compared to patients with a BD-II diagnosis. In addition, BD-I patients were more often euthymic and had lower GAF scores than BD-II patients. As a group, the BD-II patients also had more depressive symptoms. Patients with BD-I performed significantly worse than patients with BD-II on verbal learning (p ≤ 0.001), verbal memory (p ≤ 0.001), and semantic fluency (p = 0.010) (Table 1). A larger proportion of the BD-I group used antipsychotic medication (χ^2^ = 14.92, p ≤ 0.001) than the BD-II group, who to a larger extent used antidepressants (χ^2^ = 12.53, p ≤ 0.001).

#### Psychotic symptoms

Patients with psychotic BD had a shorter duration of illness and had experienced fewer elevated mood episodes than non-psychotic BD patients. Non-psychotic BD patients had, in turn, more depressive symptoms, but had higher GAF scores. They also displayed a trend toward more first-degree relatives with BD (χ^2^ = 3.71, p = 0.069). Patients with psychotic BD performed significantly worse than those with non-psychotic BD on verbal memory (p = 0.017) and semantic fluency (p = 0.011) (Table 2). A larger proportion of the psychotic BD group used antipsychotic medication (χ^2^ = 29.95, p ≤ 0.001) compared to the non-psychotic group, who to a larger extent used antidepressants (χ^2^ = 11.91, p = 0.001).

#### Polarity of presenting episode

Patients with a depressive presenting polarity were younger both at onset of disorder and at study entrance than those with an elevated presenting polarity. The group with a depressive presenting polarity also had experienced more depressive mood episodes. However, they performed significantly better than those with an elevated presenting polarity on verbal learning (p ≤ 0.001) and verbal memory (p ≤ 0.001) and had fever intrusions on the CVLT (p *=* 0.017) (Table 3).

#### Age at onset

The three age at onset groups also differed in current age. The earlier-onset groups had poorer PAS social and school scores, a longer duration of illness, a higher number of both depressive and elevated episodes, and more current depressive symptomatology. After controlling for multiple testing on neurocognitive measures, the three age at onset groups did not differ statistically significantly from each other (Table 4).

### Neurocognitive functioning across subgroups

Three neurocognitive measures differed statistically significantly across two or more subgroups, also after correcting for multiple testing: verbal learning, verbal memory, and semantic fluency. Since verbal learning and verbal memory were highly inter-correlated (*r* = 0.74, p ≤ 0.01) and analyses gave similar results, we only report here the results for verbal memory. To investigate the independent explanatory power of the different ways to subgroup, we performed two different multivariate analyses, one with verbal memory and one with semantic fluency as dependent variables (for details of procedure, see ‘Statistical analyses’ section above).

Possible confounders for the association between verbal memory and subgroups were age, gender, and duration of illness, entered in the first block. Age (p = 0.004) and sex (p = 0.002) significantly contributed to the model. In the second block, neither affective symptoms nor number of episodes affected verbal memory, while current psychotic symptoms (p = 0.003) did. Having a BD-I diagnosis, history of psychosis, and an elevated presenting episode were associated with poorer performance on verbal memory in the bivariate analyses, but all could not be entered in the same model due to collinearity problems. Regarding the effect of subgroups, the best model was the one containing elevated presenting episode followed by BD-I (Table 5).

**Table 5:** Hierarchical regression model for verbal memory

										CI	
										
	*R*^2^ change	*R*^2^	*F* change	p change	B	SE	β	*t*	p-value	Low	Up
**Block 1**	0.133	0.133	8.277	**≤0.001**							
Age					−0.071	0.024	−0.279	−2.927	**0.004**	−0.120	−0.023
Sex					1.417	0.452	0.231	3.138	**0.002**	0.525	2.309
Duration of illness					0.010	0.028	0.035	0.368	0.714	−0.044	0.065
**Block 2**	0.053	0.186	2.574	**0.040**							
Depressive symptoms					0.006	0.020	0.023	0.309	0.758	−0.033	0.045
Manic symptoms					0.063	0.057	0.090	1.106	0.271	−0.050	0.176
Current psychosis					−2.386	0.788	−0.249	−3.028	**0.003**	−3.942	−0.830
No. of episodes					0.006	0.008	0.064	0.0787	0.432	−0.009	0.022
**Block 3a**	0.039	0.225	7.888	**0.006**							
First presenting polarity					−1.263	0.450	−0.206	−2.809	**0.006**	−2.151	−0.375
**Block 3b**	0.020	0.206	4.023	0.047							
Diagnostic subgroup					0.502	0.250	0.159	2.006	**0.047**	0.008	0.195
**Block 3c**	0.007	0.193	1.425	0.234							
History of psychosis					−0.584	0.489	−0.094	−1.194	0.234	−1.550	0.382

Significant results (at p < 0.05) are presented in **bold**. CI = confidence interval; SE = standard error.

For the analysis of sematic fluency, age and level of education were possible confounders entered in the first block. Age (p = 0.005) and education (p = 0.010) significantly contributed to the model. In the second block, neither affective nor psychotic symptoms, nor duration of illness, nor number of episodes contributed to the model. Having a BD-I diagnosis and history of psychosis contributed to a poorer verbal fluency. Again, the model did not adequately fit both. The best model contained history of psychosis (Table 6).

**Table 6:** Hierarchical regression model for semantic fluency

										CI	
										
	*R*^2^ change	*R*^2^	*F* change	p change	B	SE	β	*t*	p-value	Low	Up
**Block 1**	0.062	0.062	5.350	**0.006**							
Age					−0.179	0.063	−0.201	−2.843	**0.005**	0.138	1.428
Education					0.783	0.299	0.185	2.617	**0.010**	−0.314	−0.043
**Block 2**	0.017	0.078	0.570	0.723							
Depressive symptoms					−0.090	0.073	−0.099	−1.227	0.222	−0.234	0.055
Manic symptoms					−0.090	0.210	−0.037	−0.427	0.670	−0.505	0.325
Current psychosis					0.649	2.930	0.019	0.222	0.825	−5.138	6.436
No. of episodes					0.038	0.029	0.113	1.303	0.195	−0.020	0.096
Duration of illness					0.000	0.108	0.000	0.001	0.999	−0.213	0.213
**Block 3a**	0.038	0.116	6.703	**0.011**							
History of psychosis					−4.621	1.785	−0.214	−2.589	**0.011**	−8.147	1.096
**Block 3b**	0.037	0.115	6.576	**0.011**							
Diagnostic subgroup					2.730	0.924	0.216	2.564	**0.011**	0.544	4.195

Significant results (at p < 0.05) are presented in **bold**. CI = confidence interval; SE = standard error.

## Discussion

The main finding is that three of the suggested subgroups (BD-I versus BD-II, history of psychosis, and presenting polarity) differed in regard to their association with aspects of neurocognitive functioning; in particular, verbal memory and semantic fluency. It has been suggested that verbal memory impairment is a BD endophenotype, as it seems to be a trait-related deficit 72 that is also present in relatives of patients with BD 73. It is of particular interest to show the impact of an elevated presenting polarity on verbal memory impairment, since few studies have explored the relationship between presenting polarity and neurocognition. In line with two previous studies, we also found indications (trend level significance) that patients with non-psychotic BD were more likely to have first-degree relatives with BD, compared to patients with psychotic BD 12, 13. Outside of the expected association between early age at onset and poor premorbid adjustment, there were surprisingly few group differences among age at onset subgroups.

In line with previous findings, there were clear group differences in verbal memory between BD-I and BD-II in favor of the BD-II group 50, 51. This may explain why BD-I patients have poorer general functioning than BD-II patients in spite of fewer clinical symptoms, since cognitive problems are associated with poor function 2, 47. BD-I patients had, on the other hand, better premorbid function than BD-II patients, possibly due to the earlier age at onset for the BD-II group. Due to a substantial overlap between BD-I and having a history of psychosis, group differences in verbal memory associated with history of psychosis to a large extent mirrored differences between BD-I and BD-II.

We also found support for a relationship between having psychotic BD and/or BD-I and impairments in semantic verbal fluency. Our results here are in line with previous findings of deficits in semantic verbal fluency in first-episode psychosis patients with a mania history 74 and deficits in both verbal fluency and verbal memory in first-episode psychotic BD 75. The overlap of subgroups makes it difficult to disentangle to what extent it is the disposition to experience manic symptoms, to experience psychotic symptoms, or some common factor predisposing to both these syndromes that is associated with neurocognitive dysfunction. However, the effects of manic and/or psychotic symptomatology on cognition seem stronger than the effects of depressive symptomatology. For instance, those with depressive onsets had more depressive episodes than those with an elevated onset, but still a better performance on verbal learning and memory as well as fewer errors in general. Also, even if patients with BD-II had more current depressive symptoms, they performed better than BD-I patients on verbal learning and memory, processing speed, and verbal fluency. This is in line with a recent study that found a positive association between number of manic episodes and poorer performance on neurocognitive tests in BD-I patients, with no significant effect of number of depressive episodes 76. On the other hand, these findings are equivocal, as the impact of residual depressive symptoms on cognitive domains of functioning has been demonstrated in other studies 10, 77.

The current findings seem to have clinical implications. First, as in patients with psychotic disorders 78, 79, many patients with BD have cognitive disturbances that could affect functioning and may benefit from strategies that enhance cognitive function, through cognitive remediation 80, 81. Secondly, if neurocognition is involved in the etiology and pathophysiology of the disorder, an increased understanding of this role may increase the understanding of the mechanism underlying the clinical picture and, in turn, the treatment of the disorder.

Taken together, the current findings suggest that there may be latent subgroups within the BD spectrum that to some extent encompass characteristics of several of the previously proposed subgroups; i.e., the combination of elevated presenting polarity, manic episodes and history of psychosis. These groups are characterized by impairment in neurocognitive function in particular verbal memory and semantic fluency.

### Limitations

The cross-sectional design limits the possibility to look for causal relationships. Information about onset characteristics is gathered retrospectively, with possible recall bias. Family history of psychiatric illness is based on interview with patients only. The comparison of several subgroups with repeated statistical analyses involves the risk of spurious findings, even if the main findings survive correction for multiple testing. Since this is a naturalistic study, we have not controlled use of medication, and differences in symptomatology between subgroups could be related to the use of different medications.

## Conclusions

The suggested BD subgroups show substantial overlap. At least three of the groups (BD-I, history of psychosis and elevated presenting polarity) appear to capture some common aspects of an underlying phenomenon that relates impairments in verbal memory to history of psychosis and impairments in semantic fluency to BD-I.
